# Thermo‐TRP regulation by endogenous factors and its physiological function at core body temperature

**DOI:** 10.14814/phy2.70164

**Published:** 2025-01-10

**Authors:** Makiko Kashio

**Affiliations:** ^1^ Department of Cell Physiology Kumamoto University Kumamoto Japan

**Keywords:** body temperature, temperature threshold, TRP channel

## Abstract

Transient receptor potential (TRP) channels with temperature sensitivities (thermo‐TRPs) are involved in various physiological processes. Thermo‐TRPs that detect temperature changes in peripheral sensory neurons possess indispensable functions in thermosensation, eliciting defensive behavior against noxious temperatures and driving autonomic/behavioral thermoregulatory responses to maintain body temperature in mammals. Moreover, most thermo‐TRPs are functionally expressed in cells and tissues where the temperature is maintained at a constant core body temperature. To perform physiological functions, the activity of each thermo‐TRP channel must be regulated by endogenous mechanisms at body temperature. Dysregulation of this process can lead to various diseases. This review highlights the endogenous factors regulating thermo‐TRP activity and physiological functions at constant core body temperature.

## 1 INTRODUCTION

Transient receptor potential (TRP) channels constitute a large family of cation channels that exhibit sequence conservation across a range of invertebrates to primates. Based on structural homologies, the TRP channel superfamily is divided into seven subfamilies: TRPV (vanilloid), TRPC (canonical), TRPM (melastatin), TRPML (mucolipin), TRPN (NOMPC), TRPP (polycystin), and TRPA (ankyrin). Most TRP channels form homotetramers with some exceptions. TRP channels typically function as nonselective cation channels that share a common structure of six transmembrane (TM) domains, with the ion‐permeating pore between the fifth and sixth TM domains. The activation of TRP channels by various chemical compounds and mechanical stimuli causes membrane depolarization and cytosolic Ca^2+^ elevation. General properties of TRP channels have been documented in early reviews (Pedersen et al., 2005; Venkatachalam & Montell, 2007).

**FIGURE 1:**
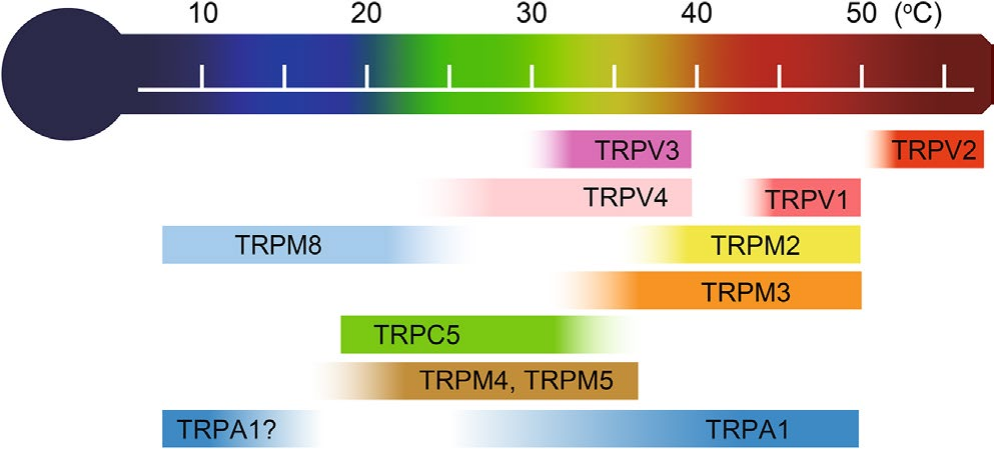
Temperature threshold for each thermo‐TRP channel.

Remarkably, several TRP channels exhibit temperature sensitivity and are therefore known as thermo‐TRPs. The TRP subfamily V type 1 (TRPV1) is the founding member of the mammalian thermo‐TRP family and is activated by high temperature (>43°C), which causes sensations of pain (Caterina et al., 1997). The discovery of TRPV1 prompted the exploration of other temperature‐sensitive molecules; to date, 11 TRP channels have been identified as thermo‐TRPs (Figure 1) (Kashio, 2021). One criterion determining temperature sensitivity is that each thermo‐TRP is activated at a characteristic temperature that represents the “threshold” for its activation. Another criterion determining temperature sensitivity is the temperature coefficient (Q_10_) value (Voets, 2012). Q_10_ is calculated using the following formula: Q_10_ = (I_T+10_)/I_T_ (where I_T+10_ = current amplitude at T + 10°C and I_T_ = current amplitude at T°C). General physicochemical reactions show Q_10_ ~ 2–3 (Bertil, 2001). Therefore, heat‐sensitive channels (Q_10_ >5) and cold‐sensitive channels (Q_10_ <0.2; 5^−1^) are particularly classified as thermosensitive TRP channels. All 11 thermo‐TRPs so far identified have been reported to meet these requirements as referenced below. Temperature change has been shown to modulate voltage‐dependent activation of thermo‐TRPs, providing a possible mechanism for the temperature sensitivity of thermo‐TRPs (Voets et al., 2004). In summary, thermo‐TRP channels show unusually high temperature dependence and cooperatively cover a wide range of temperatures from noxious cold to noxious heat (Figure 1).

The roles of thermo‐TRP functions in thermosensation (detection of external temperature) have been described in previous reviews (Dhaka et al., 2006; Kashio & Tominaga, 2022; Vriens et al., 2014). Exogenous ligands such as capsaicin (TRPV1 agonist) and menthol (TRPM8 agonist) are already known to modulate the temperature threshold for activation of their target TRPs (McKemy et al., 2002; Tominaga et al., 1998). Therefore, the current review will focus instead on endogenous mechanisms for thermo‐TRP regulation of physiological functions at the physiological core body temperature to provide a new insight into thermo‐TRP functions.

## 2 REGULATORY MECHANISMS AND PHYSIOLOGICAL/PATHOLOGICAL IMPLICATION OF THERMO‐TRPs UNDER PHYSIOLOGICAL TEMPERATURES

Table 1 shows the currently known thermo‐TRPs and characteristic temperature thresholds for the activation of each thermo‐TRP channel. Most of these thermo‐TRPs are activated by increases in temperature, whereas TRPM8 and TRPC5 are activated by decreases in temperature. In addition, most thermo‐TRPs engage in temperature sensation in primary sensory neurons and skin keratinocytes, exposed to large temperature changes from the external environment (Kashio & Tominaga, 2022). Noxious (high or low) temperatures are detected by sensory neurons that elicit defensive behavior to prevent tissue damage. In mammals, the detection of innocuous temperatures drives autonomic/behavioral thermoregulatory responses to maintain body temperature. Thermo‐TRPs are also expressed in deep organs that are continuously exposed to the core body temperature. Each thermo‐TRP channel has endogenous regulators (Table 1).

**TABLE 1:** Temperature threshold, endogenous modulators, and the expression patterns of thermo‐TRPs.

	Temperature threshold (Q_10_ value)	Endogenous regulators	Expression in sensory neurons and skin	Expression in deep organs (example)
TRPV2	>52°C (Caterina et al., 1999) (~100) (Leffler et al., 2007)	Hypotonic/mechanical stimuli (Muraki et al., 2003), reactive oxygen species (Fricke et al., 2019), steroid/cholesterol (Su et al., 2023), weak acids (Haug et al., 2024), phosphorylation (Mo et al., 2022)	Sensory neurons (Uhlen et al., 2015)	Brain, spleen, lung, immunocytes (Uhlen et al., 2015)
TRPV1	>43°C (Caterina et al., 1997) (20.6) (Welch et al., 2000)	H^+^ (Tominaga et al., 1998), phosphorylation (PKA, PKC) (Moriyama et al., 2005; Tominaga & Caterina, 2004), calmodulin (Tominaga & Tominaga, 2005), anandamide (Zygmunt et al., 1999), 1,2‐arachidonoylglycerol (Zygmunt et al., 2013), N‐arachidonoyl‐dopamine (Huang & Walker, 2006), lipoxygenase products of arachidonic acid (Hwang et al., 2000), 20‐hydroxyeicosatetraenoic acid (Wen et al., 2012), cholesterol (Liu et al., 2003)	Sensory neurons (Usoskin et al., 2015)	Brain (Martins et al., 2014), gastrointestinal tract (Yu et al., 2016)
TRPA1	<15–17°C; Moparthi et al. (2016); Story et al. (2003)	Reactive oxygen species (Andersson et al., 2008), lipid peroxide (Macpherson et al., 2007), phosphorylation (Wang, Dai, et al., 2008), Ca^2+^ (Wang, Chang, et al., 2008)	Sensory neurons (Andersson et al., 2008)	Gastrointestinal tract (Nozawa et al., 2009)
	>25°C (6) (Moparthi et al., 2016)			
TRPM3	>31–43°C, (7.2) (Vriens et al., 2011)	Pregnenolone sulfate (Vriens et al., 2011), Gβγ (Badheka et al., 2017), splicing variants (Oberwinkler et al., 2005)	Sensory neurons (Vriens et al., 2011)	Brain, kidney, pancreas (Uhlen et al., 2015)
TRPM2	>37–47°C (Kashio et al., 2012) (~100) (Kashio et al., 2022)	Adenosine diphosphate ribose (Perraud et al., 2001), Ca^2+^ (Kashio et al., 2022; McHugh et al., 2003), reactive oxygen species (Hara et al., 2002), phosphorylation (PKC) (Kashio et al., 2022)	Sensory neurons (Tan & McNaughton, 2016)	Brain, pancreas, immunocytes (Sumoza‐Toledo & Penner, 2011)
TRPM4	>16–31°C (8.5) (Talavera et al., 2005)	Ca^2+^ (Launay et al., 2002; Liu & Liman, 2003; Prawitt et al., 2003), PIP_2_ (Liu & Liman, 2003; Nilius et al., 2006), phosphorylation (PKC) (Nilius et al., 2005), calmodulin (Nilius et al., 2005)		Gastrointestinal tract, heart (Uhlen et al., 2015)
TRPM5	>16–31°C (10.3) (Talavera et al., 2005)	Ca^2+^ (Launay et al., 2002; Liu & Liman, 2003; Prawitt et al., 2003), PIP_2_ (Liu & Liman, 2003; Nilius et al., 2006), phosphorylation (PKC) (Nmarneh & Priel, 2024)		Taste cell, gastrointestinal tract (Kashio, 2021)
TRPV3	>30–39°C (Peier, Reeve, et al., 2002; Smith et al., 2002; Xu et al., 2002) (~20) (Xu et al., 2002)	Sensitization by repetitive heat stimulation (Liu & Qin, 2017), Ca^2+^‐calmodulin (Xiao et al., 2008)	Skin (Moqrich et al., 2005; Peier, Reeve, et al., 2002)	Brain, gastrointestinal tract (Uhlen et al., 2015)
TRPV4	>24–37°C (Guler et al., 2002; Liedtke et al., 2000; Watanabe et al., 2002) (10 ~ 20) (Guler et al., 2002; Watanabe et al., 2002)	Hypotonic stimuli (Liedtke et al., 2000; Strotmann et al., 2000), membrane stretch (Loukin et al., 2010), shear stress (Mendoza et al., 2010), epoxyeicosatrienoic acid (Watanabe et al., 2003), phosphorylation (PKA, PKC) (Fan et al., 2009)	Sensory neurons (Guler et al., 2002; Liedtke et al., 2000; Watanabe et al., 2002), skin (Uhlen et al., 2015)	Brain, kidney, salivary glands, vascular endothelial cell (Uhlen et al., 2015)
TRPM8	<27°C (McKemy et al., 2002; Peier, Moqrich, et al., 2002) (~0.04) (Brauchi et al., 2004)	Testosterone, PIP_2_ (Liu & Qin, 2005; Rohacs et al., 2005), Gqα (Zhang, 2019), phosphorylation (Rivera et al., 2021)	Sensory neurons (Peier, Moqrich, et al., 2002)	Prostate gland, liver (Uhlen et al., 2015)
TRPC5	<25–37°C (0.1, 37–25°C) (Zimmermann et al., 2011)	Ca^2+^ (Blair et al., 2009; Gross et al., 2009), PIP_2_‐PLC/PKC phosphorylation (Ningoo et al., 2021), Gα (Jeon et al., 2012), hypotonic/mechanical stimuli (Shen et al., 2015)	Sensory neurons (Zimmermann et al., 2011)	Odontoblasts (Bernal et al., 2021), aortic baroreceptor (Lau et al., 2016), brain, liver (Uhlen et al., 2015)

TRPV2 has a high temperature threshold (>52°C) (Caterina et al., 1999) and Q_10_ value (~100) (Leffler et al., 2007). TRPV2 is broadly expressed in various organs kept at the core body temperature (Uhlen et al., 2015). In addition to its temperature sensitivity, TRPV2 is activated by hypotonic stimuli (Muraki et al., 2003) and membrane stretch. Stretch‐induced TRPV2 activation enhances axon outgrowth of sensory and motor neurons (Shibasaki et al., 2010). TRPV2 expression is high in embryonic DRG neurons and decreases at birth, indicating that mechanical TRPV2 activation is involved in neural outgrowth during a confined stage of neural development. The gastrointestinal (GI) tract is intensely exposed to continuous mechanical stimuli. Mechanosensitive TRPV2 is involved in intestine motilities (Mihara et al., 2010). TRPV2 functions are also regulated by the endogenous factor cholesterol, which comprises 20%–30% of membrane lipid. Cholesterol binds to the transmembrane region of TRPV2 and inhibits TRPV2 activation by a synthetic activator 2‐aminoethoxydiphenyl borate (Su et al., 2023). In contrast, a cholesterol derivative, estradiol (E3), potentiates TRPV2 activity by acting on the same cholesterol‐binding site. Moreover, reactive oxygen species (ROS) sensitize TRPV2 to temperature through methionine oxidation, thereby enabling its activation at the physiological temperature (Fricke et al., 2019). A more recent paper has revealed that TRPV2 is activated and sensitized by weak acids, such as acetic acid (Haug et al., 2024). Acetic acid (pH 5.0) activates TRPV2 and sensitizes it to other TRPV2 activators. Acetic acid acts on the cytosolic face of TRPV2 in an un‐ionized state that can freely pass through the plasma membrane. However, TRPV2 sensitization is replicated by neither acetic acid at neutral pH nor the intracellular application of a strong acid (HCl). These results suggest that TRPV2 sensitization by weak acids could be mediated by a complex mechanism, not simply by binding of H^+^ or acetic acid to the cytosolic region of TRPV2. Interestingly, acetic acid sensitizes TRPV2 to mild heat stimulation (~40°C), indicating that the temperature sensitivity of TRPV2 may be modulated by weak acids. TRPV2 sensitization has also been observed with endogenous weak acids, lactic acid, and CO_2_ generated by metabolism. Moreover, tyrosine phosphorylation of TRPV2 has been shown to lower the temperature threshold (Mo et al., 2022). Therefore, TRPV2 activities at physiological/pathological conditions are potentially regulated by endogenous modulators. However, the effects of TRPV2 temperature sensitivity on its physiological functions remain largely unknown.

TRPV1 is the founding member of mammalian thermo‐TRPs and is activated by noxious high temperatures (>43°C) that cause sensations of pain (Caterina et al., 1997). The Q_10_ value above the TRPV1 temperature threshold was shown to be 20.6 in Xenopus oocytes expressing rat TRPV1 (Welch et al., 2000). TRPV1 is well known as a capsaicin receptor since it is activated by capsaicin, an ingredient of hot chili peppers (Caterina et al., 1997). Moreover, TRPV1 is activated by protons (H^+^), which are elevated in inflamed tissues (Tominaga et al., 1998). TRPV1 is functionally expressed in the peripheral endings of myelinated and unmyelinated small‐diameter sensory neurons (Usoskin et al., 2015). TRPV1 activators work synergistically to enhance pain sensation. Dorsal root ganglion (DRG) neurons from TRPV1KO (knockout) mice have a largely abrogated sensitivity to noxious heat or acid (pH 5.0); these neurons completely lacked the response to capsaicin (Caterina et al., 2000). In those TRPV1‐deficient mice, the defensive behavior induced by TRPV1‐activating stimuli was attenuated. The pathological involvement of TRPV1 in inflammatory pain is mediated by various endogenous inflammatory agents, such as bradykinin, adenosine triphosphate (ATP), and prostaglandin E2 (PGE2) (Moriyama et al., 2005; Tominaga & Caterina, 2004). When these agents bind to their receptors, TRPV1 is phosphorylated  by protein kinase A (PKA) and protein kinase C (PKC) activity. TRPV1 phosphorylation reduces the temperature threshold for TRPV1 activation to the skin temperature range, leading to sustained TRPV1 activation and chronic pain. Calmodulin binding to the N‐ and/or C‐terminal region is involved in TRPV1 desensitization (Tominaga & Tominaga, 2005), which in turn is involved in the analgesic effects of capsaicin on chronic pain along with ablation of TRPV1‐expressing nerve terminals (Simone et al., 1998). TRPV1 is activated by endocannabinoids such as arachidonylethanolamide (anandamide) (Zygmunt et al., 1999) and 1‐, 2‐arachidonylglycerol (Zygmunt et al., 2013), and by N‐arachidonoyl‐dopamine (Huang & Walker, 2006), 20‐hydroxyeicosatetraenoic acid (Wen et al., 2012), and lipoxygenase products of arachidonic acid (Hwang et al., 2000). TRPV1 activation in sensory neurons mediates the vasodilatory action of endocannabinoids. Moreover, cholesterol elevates the temperature threshold for TRPV1 activation (Liu et al., 2003). In addition to TRPV1 expression in sensory neurons, TRPV1 is found in the GI tract (Yu et al., 2016) and in various regions of the central nervous system (CNS) throughout the telencephalon, brain stem, and cerebellum (Martins et al., 2014), although some discrepancies have been observed in the expression pattern among studies. TRPV1 in the CNS is involved in pain modulation, addictive behavior, anxiety/fear, and memory learning (long‐term potentiation/depression) (Martins et al., 2014). It is worth mentioning that TRPV1 cannot be exposed to noxious high temperatures (>43°C) in the GI tract and CNS. Therefore, at core body temperature, endogenous factors may regulate the physiological functions of TRPV1.

TRPA1 is a non‐selective cation channel that is sensitive to ROS and is highly expressed in sensory neurons (Andersson et al., 2008). In addition to ROS, TRPA1 is activated by various stimuli, such as formalin, lipid peroxide, and air pollutants, which covalently modify and damage biological components such as proteins and DNA (Macpherson et al., 2007). Besides covalent modification, TRPA1 is also activated through non‐covalent mechanisms (Liu et al., 2021). TRPA1 activation in sensory neurons causes pain, which drives defensive behavior to prevent damage caused by the harmful stimuli. TRPA1 was initially reported to be cold (<17°C) sensitive (Story et al., 2003), but results of later studies are mixed. While cold sensitivity of TRPA1 has been reported in rats and mice, it was not observed in humans and monkeys (Chen et al., 2013). Double KO mice lacking both TRPA1 and TRPM8 showed no difference in cold temperature preference and avoidance behavior compared with TRPM8KO mice (Knowlton et al., 2010), suggesting a minor contribution of TRPA1 in the perception of cold temperatures. In contrast, purified human TRPA1 sorted in a planar lipid bilayer showed a clear U‐shaped cold‐ and heat‐evoked activation curve with Q10 value of 6 (>25°C), suggesting that TRPA1 has intrinsic cold as well as heat sensitivity of TRPA1 (Moparthi et al., 2016). The cold sensitivity of TRPA1 reportedly depends on its C‐terminal domain instead of its N‐terminal ankyrin domain (Moparthi et al., 2022); however, the mechanism for its cold‐sensing property remains to be further elucidated. Another study using KO mice showed that TRPA1 is involved in noxious heat sensing in primary sensory neurons, along with TRPV1 and TRPM3 (Vandewauw et al., 2018). Hydrogen peroxide (H_2_O_2_) elevates TRPA1‐dependent heat‐evoked responses in DRG neurons (TRPV1/TRPM3 double KO mice) and heat‐evoked activation of TRPA1 heterologously expressed in Chinese hamster ovary (CHO) cells. These results suggest that the cellular environment can modulate the temperature sensitivity of TRPA1. Moreover, TRPA1 function is regulated by other endogenous factors. TRPA1 phosphorylation by PKA and PKC potentially augments its activation (Wang, Dai, et al., 2008). Ca^2+^ ions both potentiate and inactivate TRPA1 activity (Wang, Chang, et al., 2008). In addition to its expression in sensory neurons, TRPA1 is expressed in enterochromaffin cells to regulate gut motility (Nozawa et al., 2009). However, the endogenous mechanisms involved in the TRPA1‐mediated regulation of gut motility remain unclear.

TRPM3 is activated by steroid hormone and high temperature, and functions as a heat sensor in primary sensory neurons (Vriens et al., 2011). TRPM3 activity increases with temperature elevation from 31°C to 43°C, with a Q_10_ value of 7.2. The neurosteroid pregnenolone sulfate (PS) is an endogenous TRPM3 regulator (Wagner et al., 2008). The synergistic effects of temperature and PS enable TRPM3 activation at body temperature with much lower PS concentrations (100 nM) than the room temperature EC_50_ (~23 μM) (Vriens et al., 2011). TRPM3 is highly co‐localized with TRPV1 and TRPA1 in sensory neurons and detects high noxious temperatures that elicit defensive behavior. Moreover, TRPM3 activity is inhibited by G‐protein βγ subunits (Gβγ) when they dissociate from the Gα subunit upon G protein‐coupled receptor (GPCR) activation (Badheka et al., 2017). In contrast, neither the Gαi nor Gαo subunits affect TRPM3 function. The inhibitory action of Gβγ mediates TRPM3 inhibition by μ‐opioid (μOR) and cannabinoid (CB1) receptor activation, indicative of antinociceptive receptor function (Dembla et al., 2017; Quallo et al., 2017). These results suggest that TRPM3 function is regulated downstream of GPCR regardless of stimulatory or inhibitory inputs. Another characteristic feature of TRPM3 is the abundance of splicing variants (Oberwinkler et al., 2005). The TRPM3 gene is composed of 28 exons and several large introns, and alternative splicing generates many variants with distinct properties such as ion permeability, sensitivity to agonists, and inhibition by Gβγ binding (Held & Toth, 2021). Moreover, TRPM3 can form heteromeric channels that generate multiple TRPM3 tetramer combinations, further increasing variation in TRPM3 function. In addition to expression in sensory neurons, TRPM3 is expressed in the brain, kidney, and pancreas, further suggesting that TRPM3 activity is potentially modulated through endogenous mechanisms at core body temperature. In addition, TRPM3 is highly and broadly expressed in the CNS, including in cerebellar Purkinje cells (Zamudio‐Bulcock et al., 2011). Several TRPM3 mutations are associated with brain dysfunction conditions such as intellectual disability and epilepsy as well as developmental and epileptic encephalopathy (DEE) (Burglen et al., 2023; Van Hoeymissen et al., 2020; Zhao et al., 2020). Many of these mutations cause gain‐of‐function changes in the TRPM3 channel. Moreover, DEE‐associated mutations in TRPM3 increase temperature sensitivity (Zhao et al., 2020). However, TRPM3KO mice exhibit no obvious changes in CNS function except for impaired pupillary light responses (Hughes et al., 2012) and peripheral heat sensitivity (Vriens et al., 2011). Overall, the physiological functions of TRPM3 in the normal CNS remain largely unknown.

TRPM2 is a non‐selective cation channel expressed in a wide variety of cell types (Sumoza‐Toledo & Penner, 2011). TRPM2 is activated by ROS (Hara et al., 2002), the intracellular endogenous agonist adenosine diphosphate ribose (ADPR) (Perraud et al., 2001), and cytosolic Ca^2+^ ions (McHugh et al., 2003). Due to ROS sensitivity, TRPM2 involvement in cell death has been extensively studied. ROS‐mediated TRPM2 activation occurs through an indirect mechanism in which ROS increase induces ADPR generation in intracellular organelles (nucleus and mitochondria) (Di Lisa et al., 2001; Schreiber et al., 2006) and through the direct action of ROS, which oxidizes a methionine residue of TRPM2 (Kashio et al., 2012). The temperature threshold for TRPM2 activation is ~47°C in heterologous expression in HEK293T cells (Kashio et al., 2012). H_2_O_2_ decreases that threshold to the physiological temperature range (<37°C), even in excised membrane patches without intracellular organelles.

An elevation in temperature from 14°C to 40°C increases TRPM2 sensitivity to ADPR and cytosolic Ca^2+^ (Bartok & Csanady, 2022). Moreover, extracellular Ca^2+^ influx into the cytosol is also elevated at high temperatures, creating a higher Ca^2+^ concentration at the intracellular Ca^2+^ binding site located just beneath the activated pore. This creates a feedback loop that further increases the temperature sensitivity of TRPM2.

**FIGURE 2:**
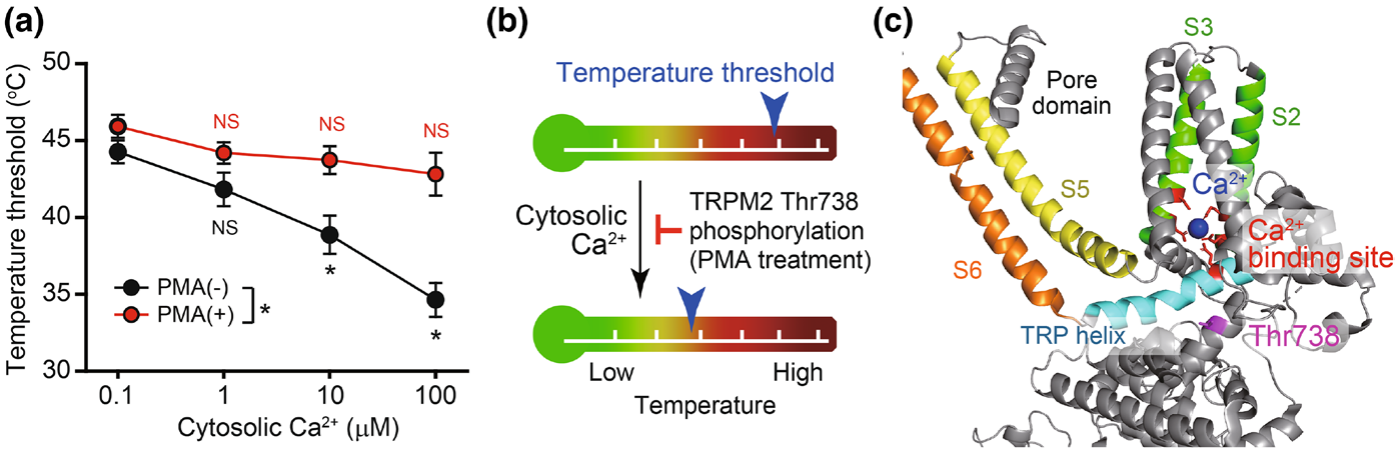
Counteraction of cytosolic Ca^2+^ and PKC‐mediated phosphorylation in regulation of temperature threshold for TRPM2. (a) Effect of PMA and cytosolic Ca^2+^ on temperature threshold for TRPM2. (b) TRPM2 phosphorylation counteracts the effect of cytosolic Ca^2+^ to elevate temperature threshold. (c) Predicted location of Thr738 in the tertiary structure of mouse TRPM2. (Adapted from DOI; 10.1113/JP283350).

In addition, cytosolic Ca^2+^ lowers the temperature threshold of TRPM2 activation in a concentration‐dependent manner (Figure 2a, black), suggesting that TRPM2 is regulated by the signals that mobilize cytosolic Ca^2+^. Q_10_ values increased ~100‐fold after heat‐evoked activation of TRPM2 under fixed cytosolic Ca^2+^ concentrations of 1, 10, and 100 μM, as well as in the absence of extracellular Ca^2+^ (Kashio et al., 2022). These results indicate intrinsic temperature sensitivity of TRPM2 without temperature‐dependent changes in the cytosolic Ca^2+^ concentration.

Interestingly, PKC‐mediated TRPM2 phosphorylation reversed the effect of cytosolic Ca^2+^ on the TRPM2 threshold (Figure 2a, red), suggesting that increasing cytosolic Ca^2+^ led to negative feedback regulation. The threonine residue (Thr738) responsible for threshold regulation by PKC is close to the Ca^2+^‐binding site near the transmembrane region of TRPM2. Phosphorylation of Thr738 would add negatively charged bulky side chains positioned to lower cytosolic Ca^2+^ affinity for the site, negating its effects (Figure 2b,c). In contrast, acidification inhibits TRPM2 activity (Du et al., 2009).

TRPM2 is expressed in sensory neurons (Tan & McNaughton, 2016); however, most TRPM2 expression is in other cells and tissues at core body temperature, including the brain, pancreas, and immunocytes (Sumoza‐Toledo & Penner, 2011). Therefore, TRPM2 activity is thought to be regulated both by temperature and by the endogenous factors at constant core body temperatures. TRPM2 is highly expressed in the CNS, including in neurons and glial cells. Most reports on TRPM2 indicate deleterious effects of TRPM2 that enhance cell death in various CNS pathological models (Malko & Jiang, 2020). However, TRPM2 is also involved in physiological functions such as body temperature regulation at the levels of central and peripheral thermoreceptors. TRPM2 is functionally expressed in warmth‐sensitive neurons in the preoptic area (POA) of the hypothalamus. Specific activation of TRPM2‐expressing POA neurons causes hypothermia with vasodilation of the tail vein and decreased locomotor activity, and attenuates fever response to PGE2 injection into POA (Song et al., 2016). Therefore, TRPM2 seems to play a role in regulating body temperature and limiting the development of fever. Considering the role of TRPM2 as a warmth sensor in the POA, the temperature sensitivity of TRPM2 is considered to be augmented by endogenous mechanisms to detect slight temperature increases within the range of brain temperature.

**FIGURE 3:**
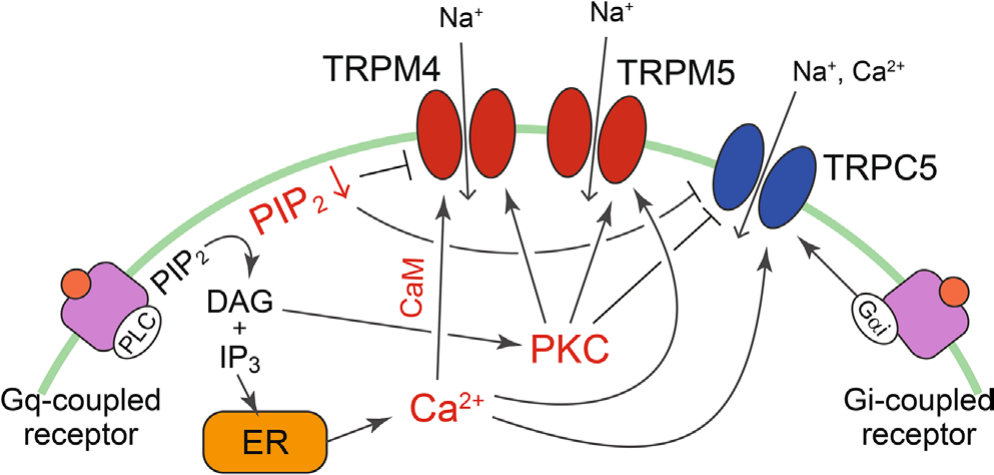
Regulatory machinery of TRPM4, TRPM5, and TRPC5 by endogenous modulators.

TRPM4 and TRPM5 are non‐selective monovalent cation channels that are activated by cytosolic Ca^2+^ (Launay et al., 2002; Liu & Liman, 2003; Prawitt et al., 2003). Both channels are activated by a temperature increase within 16–31°C (Q_10_ = 8.5 for TRPM4, Q_10_ = 10.3 for TRPM5), without a clear temperature threshold (Talavera et al., 2005). TRPM4 is highly expressed in the GI tract and heart (Uhlen et al., 2015) and is associated with cardiac electrical activity and abnormalities (Pironet et al., 2024). In contrast, TRPM5 is preferentially expressed in the chemosensory system (taste and tuft/blush cells) and plays a role in signal transduction following the activation of G protein‐coupled taste receptors (Kashio, 2021). In taste cells, the activation of taste receptors for sweet, bitter, and umami tastes leads to the activation of phospholipase C (PLC) to hydrolyze PIP_2_ to DAG and IP_3_, which then stimulates Ca^2+^ release from the endoplasmic reticulum. TRPM5, which is activated by the resulting cytosolic Ca^2+^ elevation, depolarizes the membrane potential to increase the action potential frequency and release the neurotransmitter ATP release from taste cells (Liu & Liman, 2003). Both TRPM4 and TRPM5 are regulated by PIP_2_ (Liu & Liman, 2003; Nilius et al., 2006) (Figure 3). PIP_2_ depletion following Gq‐coupled receptor activation decreases TRPM4 and TRPM5 activity, whereas PIP_2_ replenishment partly restores desensitized TRPM4 and TRPM5. Moreover, Ca^2+^‐sensitivity of TRPM4 is enhanced by PKC‐mediated TRPM4 phosphorylation and calmodulin (Nilius et al., 2005). PKC activity also enhances TRPM5 function (Nmarneh & Priel, 2024). Considering their regulation by cytosolic Ca^2+^, PIP_2_, and PKC, the activities of TRPM4 and TRPM5 at physiological temperatures are thought to be regulated downstream of signals mobilizing cytosolic Ca^2+^ ions and Gq‐coupled receptor activation.

TRPV3 is a non‐selective cation channel activated by warm temperatures (30–39°C) (Peier, Reeve, et al., 2002; Smith et al., 2002; Xu et al., 2002) with a Q_10_ value of ~20 (Xu et al., 2002). Repeated heat stimuli cumulatively augment TRPV3 activation (Peier, Reeve, et al., 2002; Xu et al., 2002) and decrease the temperature threshold for TRPV3 activation (Liu & Qin, 2017). Such TRPV3 sensitization is potentially caused by a mechanism in which repetitive heat stimuli progressively weaken the inhibitory effects of Ca^2+^‐calmodulin on TRPV3 (Xiao et al., 2008). TRPV3 is involved in warmth perception; however, peripheral thermosensation by TRPV3 appears to occur in skin keratinocytes rather than in sensory neurons (Moqrich et al., 2005; Peier, Reeve, et al., 2002). Upon TRPV3 activation by warm temperatures, skin keratinocytes release endogenous mediators such as ATP (Mandadi et al., 2009), PGE2 (Huang et al., 2008), and nitric oxide (Miyamoto et al., 2011). These molecules may activate their respective receptors in sensory neurons to mediate thermosensation. TRPV3 expression has also been observed in the CNS and gut (Uhlen et al., 2015). The endogenous factors that regulate TRPV3 are largely unknown.

TRPV4 is a non‐selective cation channel with sensitivity to warm temperatures. The temperature threshold for TRPV4 has been reported to be 24–37°C with variations (Guler et al., 2002; Liedtke et al., 2000; Watanabe et al., 2002), suggesting that the observed value is affected by experimental conditions. TRPV4 shows high Q_10_ value (~10–20) after its activation (Guler et al., 2002; Watanabe et al., 2002). Temperature sensitivity of TRPV4 is involved in perceiving innocuous warmth at sensory neurons. Warmth sensing by TRPV4 does not elicit prompt responses such as defensive behavior. However, in a thermal gradient test and two temperature‐choice test, TRPV4KO mice showed a preference for warmer temperatures, suggesting that TRPV4 plays an adaptive role in warm temperature sensation (Lee et al., 2005). In addition to warm temperatures, TRPV4 is activated by hypotonic stimuli (Liedtke et al., 2000; Strotmann et al., 2000), membrane stretch (Loukin et al., 2010), shear stress (Mendoza et al., 2010), and the endogenous arachidonic acid metabolite epoxyeicosatrienoic acid (EET) (Watanabe et al., 2003). The mechanosensitivity of TRPV4 is reportedly achieved not by directly sensing membrane stretch but indirectly by EET production accompanying mechanical stimuli (Vriens et al., 2004; Watanabe et al., 2003). Moreover, hypotonic TRPV4 activation is enhanced by PKA‐ and PKC‐mediated TRPV4 phosphorylation (Fan et al., 2009). TRPV4 is expressed in the kidneys, salivary glands, vascular endothelial cells, and skin keratinocytes (Uhlen et al., 2015), as well as in sensory neurons (Liedtke et al., 2000). TRPV4 plays a significant role in temperature‐dependent skin barrier formation (Kida et al., 2012), saliva secretion (Derouiche et al., 2018), stretch‐dependent bladder function (Gevaert et al., 2007), and shear stress‐induced vasodilation (Kohler et al., 2006). TRPV4 is also functionally expressed in the CNS and is involved in the regulation of neural activity (Shibasaki et al., 2007) and microglial motility (Nishimoto et al., 2021) by brain temperature. Therefore, TRPV4 activity is regulated by endogenous factors (physiological temperatures and mechanical stimuli) in turn regulate various physiological functions.

TRPM8 is activated by temperature decreasing below 27°C (McKemy et al., 2002; Peier, Moqrich, et al., 2002) and testosterone (Asuthkar et al., 2015). Q_10_ of cold‐evoked activation of TRPM8 is ~0.04 (23.8^−1^) (Brauchi et al., 2004). TRPM8 is expressed in a small portion of sensory neurons separately from TRPV1‐positive cells (Peier, Moqrich, et al., 2002). TRPM8 is sensitive to menthol and icilin, both of which trigger the sensation of coolness (McKemy et al., 2002; Peier, Moqrich, et al., 2002). Cool temperatures, menthol, and icilin synergistically enhance TRPM8 activation and cool sensations (Voets et al., 2004). Menthol increases the temperature threshold for TRPM8 activation (McKemy et al., 2002; Peier, Moqrich, et al., 2002). Slight decreases from the body temperature range (e.g., 35°C) can lead to considerable cytosolic Ca^2+^ increase and spontaneous high‐frequency action potential in trigeminal ganglion neurons, in contrast to TRPM8‐expressing hippocampal neurons, which show activation in response to much lower temperatures (~27°C) (de la Pena et al., 2005), suggesting that cellular environments affect TRPM8 temperature sensitivity. Indeed, on‐going activity of TRPM8 at skin temperature was inhibited by temperature increase to be involved in warmth perception (Paricio‐Montesinos et al., 2020). TRPM8 is highly expressed in the prostate ganglia and liver, as well as in sensory neurons (Uhlen et al., 2015). TRPM8 activity is also regulated by a membrane lipid, PIP_2_ (Liu & Qin, 2005; Rohacs et al., 2005). PIP_2_ depletion caused by PLC activity reduces TRPM8 activity, which is then recovered by PIP_2_ replenishment. Moreover, Gqα directly inhibits TRPM8 (Zhang, 2019). Interestingly, Gqα deletion increases the temperature threshold for cold‐evoked responses in DRG neurons. Phosphorylation of TRPM8 reportedly decreases the temperature threshold and downregulates TRPM8 function through changing the voltage dependence of its activation (Rivera et al., 2021). These results suggest that TRPM8 activity is synergistically downregulated by PIP_2_ depletion, Gqα binding, and phosphorylation following activation of the Gq‐coupled receptor.

TRPC5 is a member of the TRPC family (TRPC1‐7). The TRPC family forms functional channels via homo‐ and hetero‐multimerization of TRPC subtypes (Hofmann et al., 2002). Activity of the homomeric TRPC5 channel is elevated by a temperature decrease from 40°C to 14°C with a Q_10_ value of 0.1 (10^−1^, 37–25°C) (Zimmermann et al., 2011). TRPC5 has a relatively restricted expression pattern, including brain and liver (Uhlen et al., 2015). Even though TRPC5 is expressed in sensory neurons, its involvement in noxious cold temperature detection is unclear, because TRPC5KO mice have no obvious change in temperature preference in the range of 5–30°C or avoidance behaviors in response to noxious cold (0°C) (Zimmermann et al., 2011). TRPC5 is involved in lysophosphatidylcholine‐induced pathological pain (Sadler et al., 2021). These results suggest that the role of TRPC5 in noxious cold sensing may be altered under pathological conditions. Moreover, cold‐evoked activation of TRPC5, as well as TRPA1, has been observed in non‐neuronal odontoblasts, suggesting TRPC5 involvement in cold‐induced tooth pain (Bernal et al., 2021). Endogenous factors that regulate TRPC5 activity include Ca^2+^, PIP_2_, and the Gα subunit (Figure 3). TRPC5 is a nonselective cation channel that is Ca^2+^ permeable. TRPC5 activity is strongly augmented by intracellular Ca^2+^ elevation, mediated by TRPC5 or other pathways that mobilize Ca^2+^ (Blair et al., 2009; Gross et al., 2009). PIP_2_ along with PLC and PKC affects TRPC5 activity (Ningoo et al., 2021). PIP_2_ depletion and PKC phosphorylation of TRPC5 cooperatively inhibit TRPC5 activation, suggesting that TRPC5 regulation occurs downstream of Gq‐coupled receptor activation. In contrast, while the Gαi subunit increases TRPC5 activity, the Gβγ subunit does not (Jeon et al., 2012). These results suggest that TRPC5 activity at physiological temperatures could change with the signal input into the cell. Moreover, TRPC5 has been shown to be mechanosensitive and activated by hypotonic solution (Shen et al., 2015). TRPC5 is reportedly expressed in aortic baroreceptors and is involved in the baroreflex (Lau et al., 2016). In addition, TRPC5 is predominantly expressed in the CNS (Uhlen et al., 2015) and is involved in neuronal functions, including neurite outgrowth (Greka et al., 2003). However, further studies are required to clarify the physiological function of TRPC5, which is regulated by endogenous factors at physiological temperatures.

## 3 CLOSING REMARKS

Thermo‐TRPs in peripheral sensory neurons and skin play significant roles in thermosensation, enabling defensive behavior in response to noxious temperatures and adequate body temperature regulation in response to innocuous environmental temperature changes (Kashio & Tominaga, 2022). Studies have been discovering how thermo‐TRPs are regulated and how they exert physiological functions at the constant core body temperature. The growing knowledge of these regulatory mechanisms may prove useful in discovering novel approaches to treat diseases caused by functional abnormalities of thermo‐TRPs. Moreover, the recent development of cryo‐electron microscopy analysis has led to progress in clarifying the TRP channel structure. Rapid freezing after warming has enabled structure analysis of thermosensitive TRP channels at warm temperatures (Hu et al., 2024; Kwon et al., 2021; Nadezhdin et al., 2021). Further studies are awaited to reveal the molecular mechanisms of thermo‐TRP activity regulation at the core body temperature.

## FUNDING INFORMATION

No funding information provided.

## ETHICS STATEMENT

None.

## Supporting information


**Figure S1.** Temperature threshold for each thermo‐TRP channel.
