# Supplementary figures and images for: Thermo‐TRP regulation by endogenous factors and its physiological function at core body temperature
Source: Physiol Rep. 2025 Jan 10;13(1):e70164. doi: 10.14814/phy2.70164 (PMC11723785; doi:10.14814/phy2.70164)

Figure 1

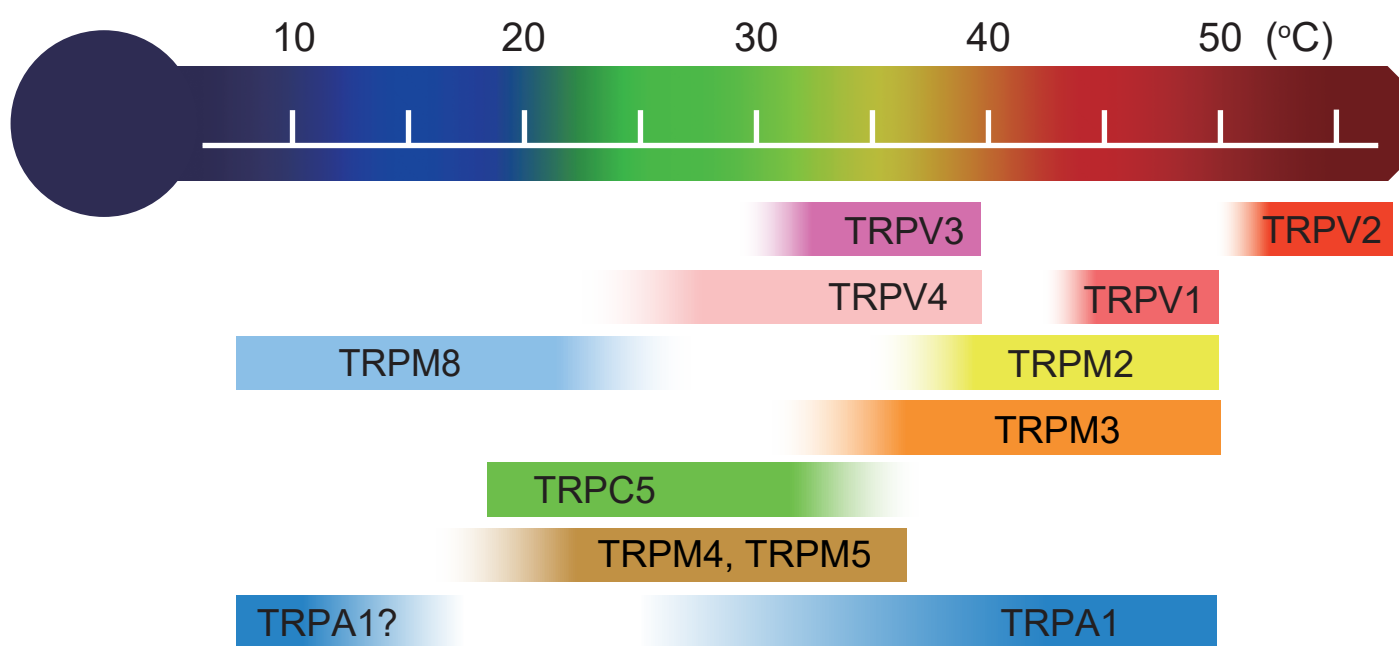

Supplement: Supplementary file 1 — Figure S1. Temperature threshold for each thermo‐TRP channel. [file PHY2-13-e70164-s001.pdf]
